# Methylation of Histone H3 on Lysine 79 Associates with a Group of Replication Origins and Helps Limit DNA Replication Once per Cell Cycle

**DOI:** 10.1371/journal.pgen.1003542

**Published:** 2013-06-06

**Authors:** Haiqing Fu, Alika K. Maunakea, Melvenia M. Martin, Liang Huang, Ya Zhang, Michael Ryan, RyangGuk Kim, Chii Meil Lin, Keji Zhao, Mirit I. Aladjem

**Affiliations:** 1 Laboratory of Molecular Pharmacology, Center for Cancer Research, National Cancer Institute, NIH, Bethesda, Maryland, United States of America; 2 Systems Biology Center, National Heart, Lung, and Blood Institute, NIH, Bethesda, Maryland, United States of America; 3 InSilico Solutions, Fairfax, Virginia, United States of America; The Hospital for Sick Children and University of Toronto, Canada

## Abstract

Mammalian DNA replication starts at distinct chromosomal sites in a tissue-specific pattern coordinated with transcription, but previous studies have not yet identified a chromatin modification that correlates with the initiation of DNA replication at particular genomic locations. Here we report that a distinct fraction of replication initiation sites in the human genome are associated with a high frequency of dimethylation of histone H3 lysine K79 (H3K79Me2). H3K79Me2-containing chromatin exhibited the highest genome-wide enrichment for replication initiation events observed for any chromatin modification examined thus far (23.39% of H3K79Me2 peaks were detected in regions adjacent to replication initiation events). The association of H3K79Me2 with replication initiation sites was independent and not synergistic with other chromatin modifications. H3K79 dimethylation exhibited wider distribution on chromatin during S-phase, but only regions with H3K79 methylation in G1 and G2 were enriched in replication initiation events. H3K79 was dimethylated in a region containing a functional replicator (a DNA sequence capable of initiating DNA replication), but the methylation was not evident in a mutant replicator that could not initiate replication. Depletion of DOT1L, the sole enzyme responsible for H3K79 methylation, triggered limited genomic over-replication although most cells could continue to proliferate and replicate DNA in the absence of methylated H3K79. Thus, prevention of H3K79 methylation might affect regulatory processes that modulate the order and timing of DNA replication. These data are consistent with the hypothesis that dimethylated H3K79 associates with some replication origins and marks replicated chromatin during S-phase to prevent re-replication and preserve genomic stability.

## Author Summary

Before each cell division, cells must accurately duplicate their chromosomes. It is critical that cells coordinate the replication of DNA with the packaging of DNA into chromosomes to insure that all genetic and epigenetic information is accurately transmitted to the next generation. In eukaryotes, replication starts at multiple sites, called “replication origins,” which are distributed throughout the genome and initiate replication in a strict order to maintain genomic stability and prevent cancer. Previous studies looked at the effect of chemical modifications on histone proteins, which affect chromosome packaging, on replication but no particular histone modifications distinctly associated with replication start sites. Here, we took advantage of recent advances in whole genome sequencing to map replication origins and histone modifications for the entire DNA in human cancer cells. One of the histone modifications we tested, methylation of lysine 79 on histone H3, was remarkably enriched at a group of replication origins. Inhibiting the enzyme that catalyzes this histone modification caused some DNA to replicate more than once during a single cell cycle, suggesting that methylation of histone H3 on lysine 79 might play an important role in controlling DNA replication.

## Introduction

The ability to turn gene expression on and off is fundamental to cell cycle progression and metazoan development. Selective gene expression requires chromatin adjustments, mediated by post-translational modifications of chromatin-associated proteins such as histones. In addition to these changes in chromatin condensation, a complete copy of the entire cellular genome must be replicated during each cell cycle. Thus, cells must coordinate replication with chromatin modifications to insure that all genetic and epigenetic information is accurately transferred to the daughter cells. It is unclear how replication proceeds along with chromatin condensation and remodeling while ensuring the fidelity of the replicated genome. In most somatic cells, DNA replication starts from consistent multiple initiation sites on each chromosome and advances in a precise temporal and tissue-specific order. It is postulated that this temporal and spatial consistency reflects a tight orchestration of replication initiation events that is necessary to coordinate replication with other chromatin transactions such as transcription.

Several lines of evidence suggest that chromatin modifications play a role in coordinating replication and transcription. Mapping the locations of replication initiation events show that replication initiation sites are enriched with transcription factor binding motifs, CpG islands and G-quartets [1]–[4]. Replication preferentially starts in transcribed chromatin [5], with the highest preference observed in moderately transcribed regions [3], and associates with genomic regions exhibiting DNAse hypersensitivity and/or containing methylated CpG sequences [3]. Although many histone modifications were examined, no particular histone modification examined thus far showed a striking association with DNA replication. Further evidence for a potential role of chromatin modifications in DNA replication stems from genetic studies characterizing the determinants of replication initiation sites. Distal DNA elements, which do not start replication but are involved in chromatin remodeling, interact with replicators, which directly facilitate initiation of DNA replication (for reviews, see [6], [7]). Such interactions are required for initiation of replication at a number of loci, including a region 40 kb upstream of the human beta-globin (*HBB*) replication origin [8], the promoter of the Chinese hamster *Dhfr* locus [9], and an enhancer of the *Th2* locus [10]. In addition, replicator sequences themselves can affect chromatin structure. For example, replicators prevent transcriptional silencing [11] by facilitating an interaction between a locus control region and a chromatin remodeling complex [12]. It is likely that chromatin modifications play a role in mediating the distal interactions that determine the locations of replication initiation events and facilitate the effects of replicators on gene expression [13], yet whole-genome mapping of replication initiation sites had not pointed to any particular histone modification [3], [4], [14].

Histone H3 exhibits methylation on lysine 79 (H3K79) catalyzed by the methyltransferase DOT1 (Disruptor of Telomere silencing 1) enzyme (DOT1-like, or DOT1L in humans) that facilitates telomeric and Sir mediated silencing [15]. DOT1 promotes the mono-, di- and tri-methylation of H3K79 [16] and these methylations are involved in transcriptional elongation, DNA repair, and heterochromatin maintenance. In yeast, cell cycle regulated genes exhibit differential di- and tri-methylation of H3K79 [17], and methylation of H3K79 is required for the G1 and intra S-phase checkpoints. H3K79Me2 interacts with CAF-1 and is particularly abundant during late S-phase [18]. In mammals, methylation of H3K79 is abundant in active genes, including the murine beta-globin locus [19]. Human *DOT1L* methylates H3K79 and associates with a complex that participates in Wnt signaling [20], which includes beta-catenin, Skp1, and TRRAP. DOT1L is required for development and plays essential roles in early erythropoiesis [21] and cellular reprogramming during development [22]. Proper functioning of DOT1L, in collaboration with H2B ubiquitination, promotes the DNA damage checkpoint [23], likely by H3K79Me2 mediated targeting of 53BP1 to DNA damage lesions [24]. Methylation of H3K79 was implicated as a determinant of global genomic repair [25] and silencing of tumor suppressor in hematologic malignancies [26]. Aberrant methylation of H3K79 by DOT1L is associated with MLL rearrangements in leukemia [27]–[29], possibly by mistargeting of *DOT1L* activity [30].

The human beta-globin locus (*HBB*) contains one of the most intensely studied replication initiation regions (IRs) [31]–[34] in mammalian cells. This particular origin is used in both erythroid and nonerythroid cells, but the timing of replication initiation differs between these two cell types. In erythroid cells, the *HBB* locus initiates DNA replication during the early stages of S-phase, while initiation in non-erythroid cells typically occurs during late S-phase [35], [36]. The *HBB* IR contains two independent replicators (Rep-P and Rep-I), and each can initiate DNA replication at both native and ectopic sites [31], [33]. Detailed genetic analyses have revealed that both Rep-P and Rep-I contain an AT-rich sequence and an asymmetric purine:pyrimidine (AG) sequence, both of which are required for replication initiation [34]. The *HBB* IR, therefore, provides an excellent system to study replicator-binding proteins that both recruit the general replication machinery to specific chromatin sites and interact with the cell cycle machinery. Because of the availability of mutants that do not initiate replication, this system is ideally suited to investigate whether any particular protein-DNA interaction correlates with functional replicator activity.

Here, we asked whether methylation of H3K79 is associated with replication initiation events genome wide and followed those general observations with functional studies at the well-characterized replication initiation site within the human beta-globin locus. We also studied the function of H3K79 methylation in replication initiation by depletion of *DOT1L*. Our results suggest that H3K79Me2 is associated with initiation of DNA replication genome wide, that the modification of H3K79 at the beta globin locus correlates with replicator activity and that H3K79 methylation might play a role in ensuring that at each locus, replication would initiate only once per cell cycle.

## Results

### Chromatin containing H3K79Me2 is enriched in replication initiation events genome-wide

We have recently mapped the locations of replication initiation events genome-wide in several human cell lines. Although replication initiation events were enriched in DNAse hypersensitive sites and in methylated CpG rich regions, previous studies in our lab and others have not identified chromatin modifications that exhibited a high enrichment for replication initiation events [3]. Because replication initiation events tend to be depleted at transcription start sites and enriched just downstream of those sites [3], we asked if H3K79 dimethylation, which exhibits a similar pattern, might mark replication initiation events. We performed chromatin immunoprecipitation followed by sequencing (ChIP-Seq) with an antibody specifically directed against H3K79Me2 in human eryhtroleukemia K562 cells. K562 cells express the gamma globin in which the beta-globin locus replicates early during S phase and were used for numerous replication-related studies including whole genome origin mapping [3]. In addition, ChIP-Seq data delineating biding sites of many histone modifications are available for K562 cells, and the cells are amenable to fractionation according to cell cycle stages using centrifugal elutriation (see Table S1 for a complete list of cell lines used in this study, as well as their backgrounds and/or reasons being used). The genomic locations of chromatin enriched in H3K79Me2 were identified by massively parallel sequencing and visualized relative to the locations of replication initiation events, based on sequencing short, RNA-primed nascent DNAs isolated from K562 cells [3]. The frequency of initiation events at individual genomic regions was measured as the ratio between reads obtained from a nascent strand preparation and reads obtained from a corresponding control genomic DNA preparation. The reads were calculated as reads per kilobase (kb) per million mapped reads (RPKM). The results were visualized using the Integrative Genome Viewer 2.1 (Broad Institute).

**Figure 1:**
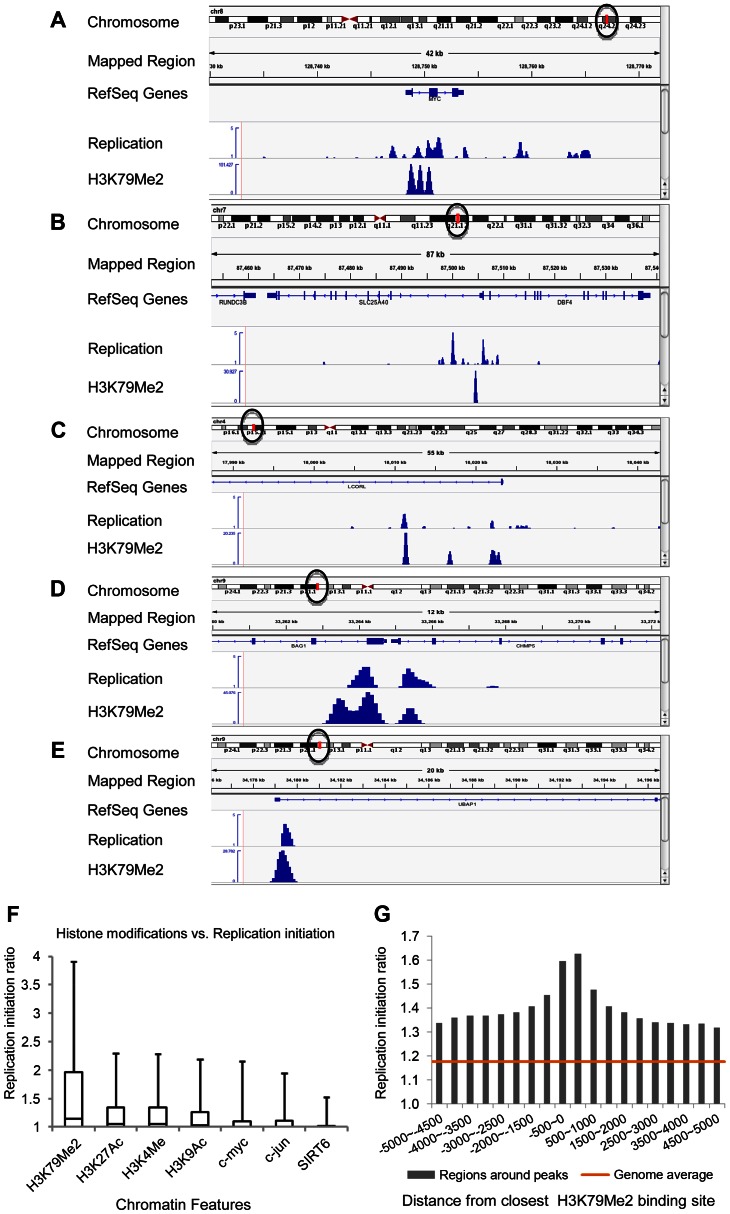
H3K79Me2 containing chromatin is associated preferentially with replication initiation sites genome-wide. A–E. Screenshots of replication initiation data visualized with the integrated Genome Viewer (http://www.broadinstitute.org/igv/). A chromosome map is shown at the top, and the region-of-interest is delineated by a circle. The analyzed region is shown underneath the ideogram, with map coordinates indicated. The Replication panel shows the distribution of replication initiation events (ratio of reads obtained from a nascent strand preparation, and reads obtained from a corresponding control genomic DNA preparation) of the region-of-interest obtained from our published database, see [3] for details. Regions abundant in H3K79Me2 immunoprecipitated chromatin from H3K79Me2 ChIP-Seq in K562 cells are shown below the initiation profile as reads per kilobase per million aligned reads (RPKM). Ref-Seq genes are aligned above the experimental data. A. The distribution of H3K79Me2 and replication initiation events in the MYC locus. B. The distribution of H3K79Me2 and replication initiation events in the DBF4 locus. C. The distribution of H3K79Me2 and replication initiation events in the LCORL locus. D. The distribution of H3K79Me2 and replication initiation events in the BAG1 locus. E. The distribution of H3K79Me2 and replication initiation events in the UBAP1 locus. F. A box plot comparing the relative enrichment of replication initiation events in chromatin featuring H3K79Me2 obtained by ChIP-Seq as described in methods with various other chromatin features as reported in the UCSC genome browser (for details, see[3]. Boxes indicate distributions of the second and third quartiles and whiskers, 95^th^ percentiles. The horizontal lines in the boxes represent medians. Values lower than a unit were converted into 1. Methylation of H3K79 exhibits a marked enrichment in replication initiation events that was higher than any other measured histone modification or transcription factor. G. A histogram showing the replication enrichment ratio (calculated as in A, B) for genomic regions as a function of their distance from the closest H3K79Me2 interaction sites. A box plot version of the same histogram is shown as Figure S1.

Figure 1A–E shows the replication initiation ratio (nascent strands *vs*. genomic RPKM) and H3K79Me2 in sample human loci. For each image, a chromosome map is shown at the top, and the region-of-interest is circled. The analyzed region is shown underneath the ideogram, with map coordinates indicated. Ref-Seq genes are aligned under the map coordinates. The replication tracks show the distribution of sequence reads (aligned with the indicated region) obtained from massively parallel sequencing of nascent strands from K562 erythroleukemia cells as described [3]. All data are shown as the ratio of reads obtained from a nascent strand preparation, and reads obtained from a corresponding control genomic DNA preparation. The y-axis indicates the nascent strand/genomic DNA ratio. The H3K79Me2 tracks indicate the distribution of reads from ChIP-Seq analyses with H3K79Me2 specific antibodies as described in the Methods section. The data show that chromatin regions exhibiting H3K79 dimethylation exhibited high enrichment for replication initiation sites, measured by the ratio of reads on nascent strands vs. genomic DNA controls. When we calculated the enrichment of genome-wide H3K79Me2 peaks obtained from the ChIP-Seq data for replication initiation events, overall, 23.39% of the H3K79Me2 peaks were detected in regions adjacent to replication initiation events whereas an association with replication initiation events was expected in only 7.26% of the size-matched randomized feature regions (Table 1, “overall”). Chromatin regions exhibiting H3K79 methylation markedly enriched in replication initiation events, showing an average whole-genome RPKM ratio of 1.8 (Figure 1F). As shown previously [3], replication initiation events were also enriched, to a lesser extent, in chromatin regions exhibiting acetylation of histone H3 on lysines 9 and 27 and methylated on lysine 4, and in chromatin binding transcription factors c-Jun and c-Myc. As shown in Figure 1F, the extent of enrichment for replication initiation events in regions exhibiting methylation of histone H3 on lysine 79 is markedly higher than in regions exhibiting other histone modifications. Given the sequencing depth and the large sample size, an average value of 1.3 (as calculated for H3K27Ac) represents significant enrichment over the average whole-genome RPKM ratio (p<10^−6^) despite the seemingly low numerical values. The statistical significance of enrichment in H3K79Me2 in replication initiation events is very high (p<10^−20^). Replication initiation events were markedly enriched in regions within 500 bp of an H3K79Me2 binding site, and this is not true for other regions such as JunB binding sites (Figure 1G and Figure S1, S2). H3K79 methylation exhibited a similar enrichment with replication initiation events as DNAse hypersensitivity and CpG methylation, however these traits did not exhibit any synergy (Figure S3).

**Table 1:** Fraction of H3K79Me2 regions that are within 2000 bp from a 15% FDR replication peak.

	H3K79Me2 regions	Average from 100 runs with size-matched randomized feature regions
Overall	23.39	7.26
G1	25.27	7.31
S	20.90	7.29
S-only*	12.25	7.29
G2	27.37	7.31

* Chromatin regions associated with H3K79Me2 solely in S-phase.

**Figure 2:**
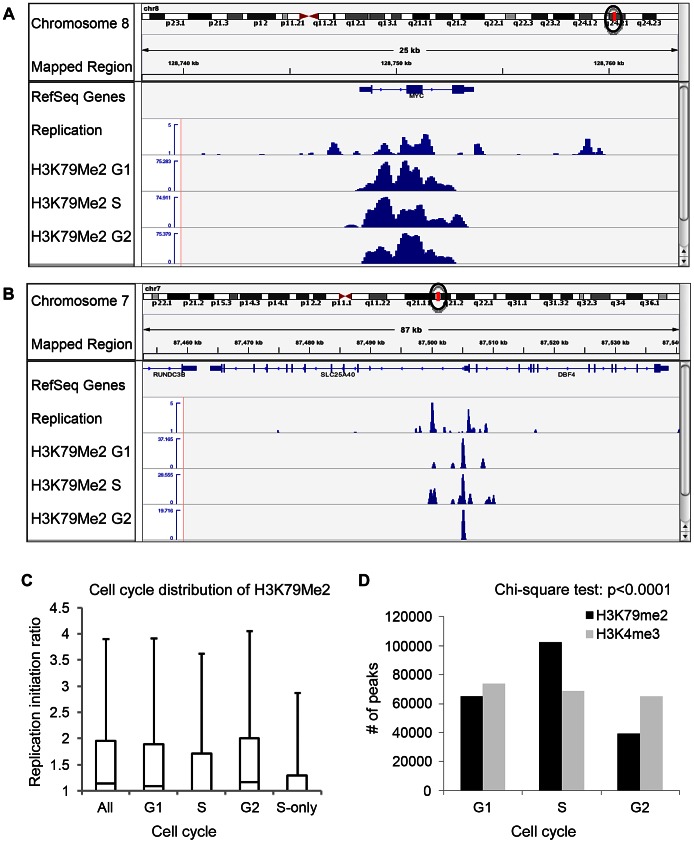
Preferential enrichment of initiation events in H3K79Me2 containing chromatin at the G1 and G2 phases of the cell cycle. An asynchronously growing population of K562 cells was fractionated into separate populations of G1, S-phase and G2/M cells using centrifugal elutriation. Chromatin from each separate cell cycle phase population was isolated and analyzed by H3K79Me2 ChIP-Seq as described in the legend to Figure 1. A. The distribution of H3K79Me2 and replication initiation events in the MYC locus during the different phases of the cell cycle. B. The distribution of H3K79Me2 and replication initiation events in the DBF4 locus during the different phases of the cell cycle. C. A box plot as described in Figure 1 legend showed the relative enrichment in H3K79Me2 for replication initiation events during the different phases of the cell cycle. Boxes indicate distributions of the second and third quartiles and whiskers, 95^th^ percentiles; values lower than a unit were converted into 1. All, replication initiation ratios in regions that were associated with H3K79Me2 in an asynchronous cell sample representing all stages of the cell cycle; G1, S and G2, regions associated with H3K79Me2 in samples from cells at the appropriate cell cycle phase; S-only, regions that were only associated with H3K79Me2 in S-phase but not in G1 and G2. D. The number of H3K79Me2 associated peaks on chromatin during subsequent stages of cell cycle progression. H3K4Me3 was used as a control. Chi-square test shows that the distributions of H3K79Me2 and H3K4Me3 in G1, S and G2 cells are statistically different (p<0.0001). H3K79 dimethylation was more abundant and exhibited a wider distribution during S-phase, but chromatin regions associated with H3K79Me2 solely in S-phase were not further enriched in replication initiation events. The association of H3K79Me2 with replication initiation events was re-established in G2.

To further explore the association between H3K79 dimethylation and replication initiation, we measured the levels of H3K79Me2 enrichment during the G1, S, and G2 phases of the cell cycle in K562 cells fractionated into synchronous cell cycle populations using centrifugal elutriation (Figure 2, Table 1, rows G1 through G2, and Table 2) and Western immunoblots. The overall level of H3K79Me2 modification did not change during the cell cycle (Figure S4A) and the abundance and distribution of chromatin regions exhibiting H3K79Me2 was similar in the G1 and G2 phases of the cell cycle. However, the distribution of H3K79Me2 associated regions expanded in S-phase. Interestingly, chromatin regions that exhibited H3K79Me2 only during S-phase did not display a high association with replication initiation events (Figure 2C and Table 1, “S only”). By contrast, another modification, H3K4Me3, did not exhibit a similar expansion of peaks in S-phase and displayed a consistent distribution throughout the cell cycle (Figure 2D and Table 2). These observations support the conclusion that the H3K79Me2 modification associates preferentially in a cell cycle specific manner with replication initiation regions.

**Table 2:** Number of 100 bp bins associated with histone H3 methylation on lysines 79 and 4 during the cell cycle*.

	G1	S	G2
H3K79me2	65284	102372	39271
H3K4me3	73851	68750	65152

* Data used for Figure 4D.

### H3K79Me2 binds a functional replicator but not a mutant that cannot initiate replication

**Figure 3:**
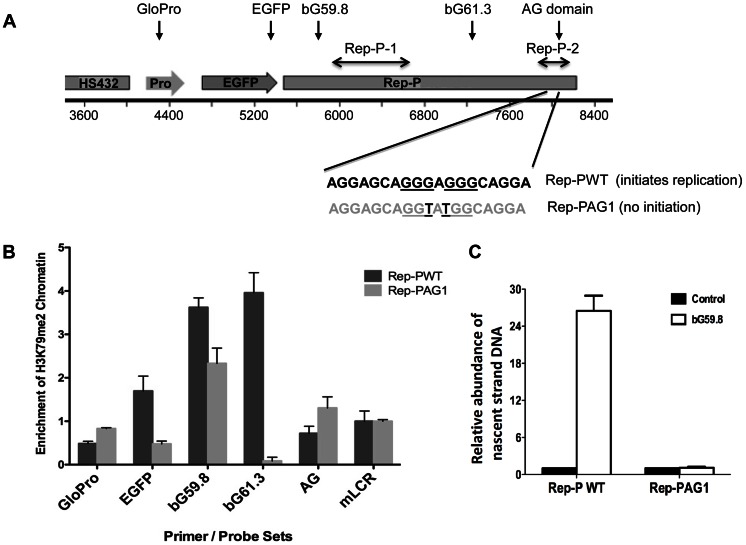
H3K79 dimethylation accompanies replicator activity. A. Two transgenes containing sequences from the human beta-globin Locus Control Region (HS432), the human beta-globin promoter (GloPro) driving enhanced green fluorescent protein (EGFP) and two variants of the Rep-P replicator were inserted into a single location on murine chromosome 15 in murine erythroleukemia (RL4) cells [11]. Murine cells were utilized to facilitate detection of the exogenous sequences from the human beta globin locus; the murine locus control region was used as a control. One transgene variant (Rep-PWT) contains the native unaltered sequence of Rep-P-2 (starting 87 bp 5′ of the beta-globin promoter) that is essential for replication initiation. The other transgene variant (Rep-PAG1) harbors two point mutations at the Rep-P-2 sequence that prevent initiation of DNA replication. B. Chromatin immunoprecipitation with antibodies directed against H3K79Me2 in RL4 cells carrying wild-type (Rep-PWT) or mutant (Rep-PAG1) transgene cassettes. Each column represents the mean enrichment value inH3K79Me2 calcualted based on real-time PCR amplification of chromatin immunoprecipitation using the indicated primer pairs. Error bars indicate standard deviations. C. Nascent strand abundance analysis in RL4 cells carrying wild type (Rep-PWT) or mutant Rep-P (Rep-PAG1) transgene cassettes. Primers and probes used, listed in Table 3, included GloPro (human beta-globin promoter), EGFP (the EGFP gene), bG59.8 (Rep-P 5′ end sequence), bG61.3 (Rep-P 3′ end sequence), AG (the AG region of Rep-P), and mLCR (murine Locus control region). All except mLCR are sequences from the transgene and their location is illustrated in the map shown in panel A. Sequences from transgenes harboring the active replicator were enriched in H3K79Me2 containing chromatin whereas sequences from the mutant transgene that did not initiate replication, were not.

To determine if H3K79 was methylated at a replicator sequence essential for initiation of DNA replication, we performed chromatin immunoprecipitation (ChIP) analysis with the Rep-P asymmetric region of the endogenous β-globin locus in K562 cells (for maps, see Figure 3A and Figure S4B). This region is essential for initiation of DNA replication from Rep-P, one of the two adjacent replicators that can initiate DNA synthesis at the β-globin locus [33], [34]. As shown in Figure S4B, probes that colocalized with the Rep-P replicator (bG59.8, bG61.3, AG) exhibited higher enrichment in H3K79Me2. We also tested the association between H3K79Me2 and late-replicating replication initiation site in Jurkat cells, which are human T-cells that do not express any gene from the human beta-globin locus and yet initiate replication from Rep-P late during the S-phase of the cell cycle. The asymmetric region (probe marked AG) exhibited a high level of H3K79 dimethylation in both K562 cells that express γ-globin and in Jurkat cells, which do not express any gene within the *HBB* cluster and replicate the entire locus late in S-phase.

We then investigated whether DNA sequences required for initiation of DNA replication were also essential for enrichment of H3K79Me2 in the Rep-P region. To that end, we introduced transgene cassettes that included the LCR, an enhanced GFP expression cassette driven by the beta-globin promoter, and Rep-P variants into a site termed random locus 4 (RL4) in murine erythroleukemia (MEL) cells. This site was used previously to assess the roles of replicator sequences in initiation of DNA replication and gene silencing [37]. The RL4 site was engineered to contain an inverted pair of LoxP sites so that any inserted transgene cassette in that locus could be exchanged with other cassettes inserted precisely at the same genomic location. This system can facilitate testing for effects of distinct mutations and sequences from the murine beta globin locus serve as controls for human inserted sequences. Previous studies have shown that the unmodified RL4 site exhibits a heterochromatin conformation and does not initiate replication, hence initiation activity detected at this site after insertion of ectopic sequences reflects sequence information encoded within the inserted transgens [37]. Using this feature, we tested a transgene cassette that included an intact Rep-P (Rep-PWT – Figure 3A) and a cassette that included a Rep-P variant with only 2 nucleotides mutated in AG region of the rep-P (Rep-PAG1). The intact Rep-P (Rep-PWT) exhibited enrichment of H3K79Me2 (Figure 3B, probes bG59.8, 61.3 – see Figure S4B and Table 3 for locations and sequences of primer pairs and probes) and initiated replication at the ectopic site (Figure 3C). By contrast, the mutant Rep (Rep-PAG1) did not exhibit enrichment of H3K79Me2 and did not initiate replication (Figure 3, B and C, respectively). Although the orientation of the transgene at the RL4 site affects transcriptional silencing [37], analyses of Rep-PAG1 yielded similar results when the transgene was inserted in both orientations (data not shown). It should be noted that in the RL4 locus the highest enrichment in the intact Rep-P transgene was observed upstream of the asymmetric region (primer pairs bG59.8 and 61.3) whereas the asymmetric locus exhibited a higher enrichment at the native locus in K562 cells (Figure S4). This shift was most likely due to the fact that the inserted transgene contained only one of the two replicators at the replication initiation region (Rep-P without Rep-I). Importantly, since of H3K79Me2 was detected in the active but not the inactive Rep-P, these observations suggest an association between H3K79 dimethylation and replicator activity.

**Table 3:** Primers and probes used in the study.

Primer/probe	sequence
hHS2 FW	GGCTCAAGCACAGCAATGC
hHS2 RE	CATCACTCTAGGCTGAGAACATCTG
hHS2 Probe	FAM-AGTCATGCTGAGGCTTAGGGTGTGTGC-TAMRA
bG59.8 FW	TGG AAA AGC AAC CCC TGC
bG59.8 RE	AAC TAT GGA TCC TTC TCT TGT GTT GG
bG59.8 probe	FAM-GCT GCA GAT ACC ATC ATC CTG GCT TCA A-TAMRA
bG61.3 FW	ACA GAG GCT TTT TGT TCC CCC
bG61.3 RE	GGT AAT CAG TGG TGT CAA ATA GGA GG
bG61.3 probe	FAM-GAC ACT CTT GCA GAT TAG TCC AGG CAG A-TAMRA
AG FW	CAACTCCTAAGCCAGTGCCAGAAG
AG RE	TGCCCTGACTTTTATGCCCAGC
AG probe	FAM- TCATCACTTAGACCTCACCCTGT-TAMRA
GloPro FW	TGAGGGTTTGAAGTCCAACTCC
GloPro RE	GGTCTAAGTGATGACAGCCGTACC
GloPro Probe	FAM- AAGCCAGTGCCAGAAGAGCCAAGGA-TAMRA
EGFP FW	AGCAAAGACCCCAACGAGAA
EGFP RE	GGCGGCGGTCACGAA
EGFP Probe	FAM-CGCGATCACATGGTCCTGCTGG-TAMRA
mLCR FW	TCATCAGTCTTGTGTGAAAGTG CTT
mLCR RE	AGTAGAGTGGAGAATCAAAAACACATTT
mLCR probe	FAM-ATCTAGTGAACCACATTAACTGGCCCTGGC-TAMRA

### H3K79Me2 plays a role in restricting replication once per cell cycle

**Figure 4:**
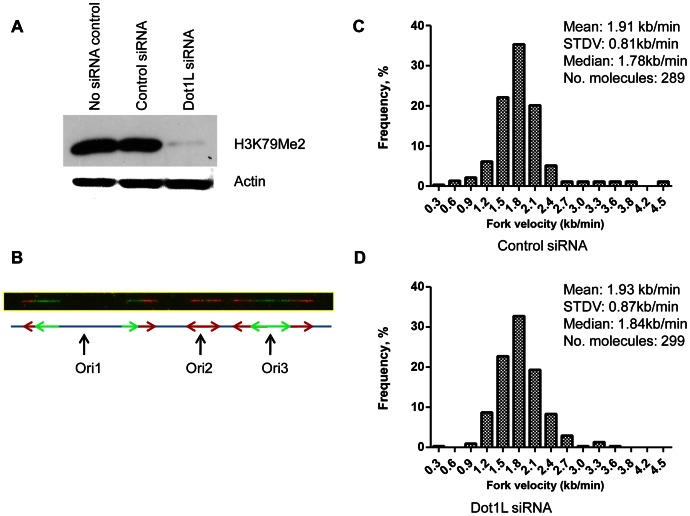
Depletion of H3K79 methyltransferase *DOT1*L does not change replication elongation and initiation rates. HCT116 cells were transfected with siRNA directed against *DOT1L* or scrambled siRNA control twice with a 48 h interval. A. Levels of H3K79Me2 in total cell proteins collected 72 hours after the second transfection. Actin was used as a loading control. B–D. Cells were labeled sequentially with ldU and CIdU as described in the Methods section. Cells were then harvested and DNA extracted. The DNA was stretched on a silanized microscope coverslip, and visualized with antibodies against DNA containing ldU and CldU [48]. B. An example of combed DNA. Replication fork progression rates were calculated from the length of CldU (red replication tracks) and IdU (green replication tracks) signals. Inter-origin distance was measured by identifying replication initiation events (ori1 to ori3). C. A histogram of the distribution of replication fork speeds measured in DNA fibers from cells transfected with scrambled siRNA. D. A histogram of the distribution of replication fork speed measured in DNA fibers from cells transfected with siRNA targeting *DOT1*L. Depletion of *DOT1*L reduced the levels of H3K79Me2 but did not affect DNA replication fork velocity and inter-origin distance (Figure S5).

That H3K79Me2 is strongly associated with the start of DNA replication implicates a causal role for H3K79 dimethylation in regulating the initiation of DNA replication. To address this question, we depleted DOT1L, the enzyme that methylates histone H3 on lysine K79. These experiments were performed in human colon cancer HCT116 cells. HCT116 cells were chosen for this analysis because these cells exhibit a relatively stable karyotype and can be easily transfected with siRNA, and are therefore suitable for analyses aimed to test the effects of siRNA mediated gene silencing on cell cycle distribution. In HCT116 cells, siRNA mediated DOT1L depletion prevented H3K79 dimethylation (Figure 4A). We used those cells to measure the rate of DNA synthesis in cells with and without DOT1L using dynamic molecular combing (Figure 4, B–D and Figure S5). As shown in Figure 4, C and D, depletion of DOT1L did not affect replication fork velocity, suggesting that H3K79 methylation does not directly affect the progression of replication forks. DOT1L depletion also did not affect the frequency of replication initiation events (Figure S6).

**Figure 5:**
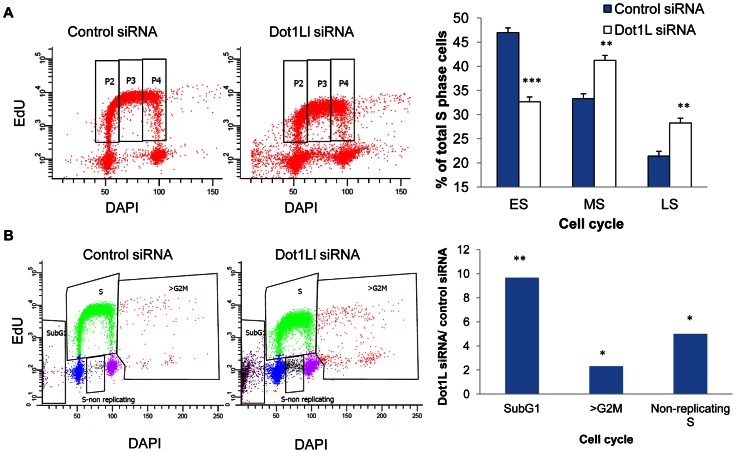
Effects of Dot1L depletion on cell cycle progression. HCT116 cells transfected with siRNA directed against *DOT1L* or scrambled siRNA control three times, first with a 48 h interval followed by a 72 hr interval. Cells were collected for cell cycle analysis by FACS 3 days after the third transfection. EdU were added to cells for 45 minutes before harvesting cells. Click-iT EdU kit from Invitrogen was used to detect replicating cells and DAPI was used to determine DNA content. A. The left panel shows a representative cell cycle profile for cells transfected with control siRNA. The middle panel shows a representative cell cycle profile for cells transfected with siRNA directed against DOT1L. The right panel shows a histogram illustrating the fraction of cells in early, middle and late S-phase (ES, MS and LS, representing the number of cells in the P2, P3 and P4 FACS gates, respectively). Two stars represent a statistically significant change with p value lower than 0.01; three stars represent a statistically significant change with p value lower than 0.001. Dot1L depletion increased the fraction of late S-phase cells. B. The left and middle panels show the cell cycle distribution of the same cells as in A, illustrating the FACS gates used to identify the sub-G1, non-replicating S and >G2/M cell populations. The histogram plots the fraction of the gated populations in DOT1L depleted cells divided by the fraction of the same gated populations in cells transfected with a control siRNA. A star represents a statistically significant change with p value lower than 0.05; two stars represent a statistically significant change with p value lower than 0.01. Table S2 shows the fraction of cells at each cell cycle phase. Dot1L depletion caused a limited increase in the fraction of apoptotic cells, cells with S-phase DNA content that did not incorporate EdU and cells with DNA content greater than G2/M.

We then asked whether cell populations in which DOT1L was depleted exhibited changes in cell cycle patterns. The overall distribution of cells in the G1, S and G2/M phases of the cell cycle was similar in control and DOT1L depleted cells, but FACS analyses indicated that DOT1L depleted cells had fewer cells in the early S-phase and more cells with late S-phase DNA content than cells in which DOT1L was not depleted (transfected with a control siRNA; Figure 5A). As shown in Figure 5B, DOT1L depletion also resulted in an increased frequency of apoptosis (subG1) and in an increase in the fraction of cells exhibiting DNA content greater than 4N (>G2/M) or cells that did not synthesized DNA despite a DNA content between 2N and 4N (S non-replicating). Similar results were observed in U2OS osteosarcoma cells (Figure S7 and Table S2). These observations suggested that although most cells can continue to proliferate and replicate DNA in the absence of methylated H3K79, prevention of H3K79 methylation might affect regulatory processes that modulate the order and timing of DNA replication.

**Figure 6:**
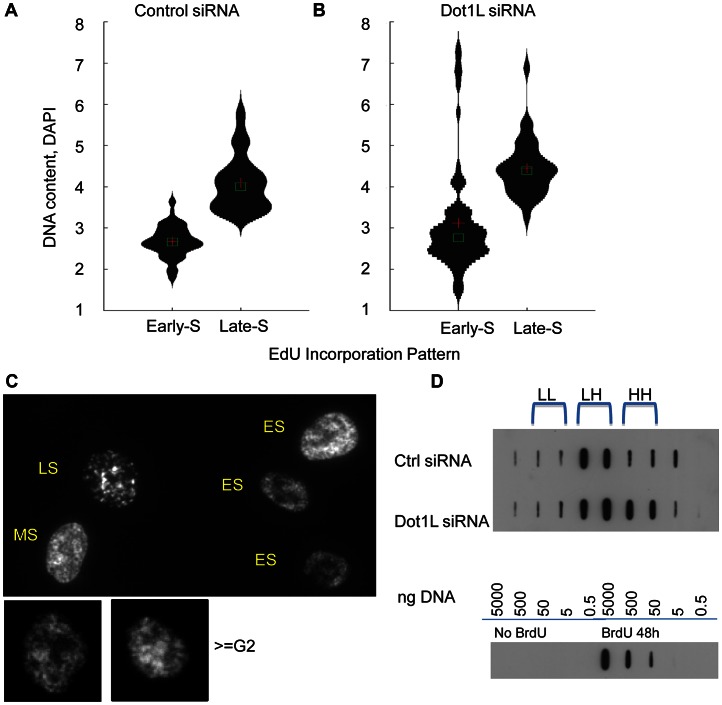
Over-replication in *DOT1*L depleted cells. HCT116 cells were transfected with siRNA directed against *DOT1L* or scrambled siRNA control twice with a 48 h interval, and collected 3 day after the second transfection. (A–C) Cells were labeled with 10 µm of EdU for 1 hour before harvest and EdU distribution patterns were visualized along with DNA content measurement using DAPI. DNA/DAPI content was quantified in cells exhibiting early and late S phase EdU distribution using the Pathway imaging system (BD). DNA content distribution in early S-phase cells and late S-phase cells determined by EdU patterns in HCT116 cells transfected with control scrambled siRNA (panel A) or *DOT1*L siRNA (panel B). Cross shows mean and square box shows median of DNA content. C. Example images for diffuse “early” EdU pattern, large punctuate structures of “late” EdU pattern and re-replicated larger cells (> = G2 by DAPI DNA content) with early EdU patterns. *DOT1*L depleted cells, but not control cells, contained a population of cells with DNA content greater than 4N exhibiting early replication patterns. D. BrdU density gradients measuring DNA re-replication. Top panel: HCT116 cells were transfected with control siRNA or Dot1L siRNA as described above and labeled with Bromodeoxyuridine (BrdU) for 18 hours before harvest. Genomic DNAs were fractionated on CsCl gradients and BrdU substituted DNA was detected using anti-BrdU antibodies on a membrane. BrdU substituted DNA is denser (heavier) than unsubstituted DNA. LL: unsubstituted DNA; HL: semi-substituted DNA; HH: fully substituted. Bottom panel: Serial dilutions of unsubstituted genomic DNA and fully BrdU substituted DNA sample (isolated from cells incubated with BrdU for 48 hours) were used as controls to evaluate the specificity of the anti-BrdU antibody.

Because H3K79 methylation was enriched in replication initiation sites, we asked whether the order of DNA replication was altered in cell populations in which DOT1L was depleted. To measure DNA replication patterns, cells were pulse-labeled with the nucleotide analog EdU for 60 min. DNA content (DAPI intensity) was measured, along with the pattern of EdU staining (Figure 6, A and B). The pattern of replication foci as exhibited by EdU incorporation was recorded for each nucleus (for examples, see Figure 6C). In untreated cells, diffuse replication foci patterns are characteristic of early S-phase (Figure 6C, ES), whereas nuclei exhibiting a few condensed replication foci are abundant in late S-phase (Figure 6C, LS). We then tallied the frequency of early and late S-phase EdU staining patterns in cell populations exhibiting early and late S-phase DNA content measured by DAPI staining. As expected we observed that diffuse patterns were indeed abundant in cells exhibiting early S-phase DNA content, whereas clustered patterns are frequent in cells exhibiting late S-phase DNA content (Figure 6A and B). However, cell populations in which DOT1L was depleted contained a small subset of cells with DNA content of more than 4N exhibiting diffuse replication patterns (Figure 6B; examples are shown in Figure 6C, > = G2M). This cell population might represent cells with late S-phase DNA content that re-replicated DNA that had already been duplicated earlier during the same S-phase, thus exhibiting an early S-phase replication pattern. This pattern is consistent with the observation that depletion of DOT1L resulted in an increased fraction of cells with DNA content larger than 4N, representing cells that skipped mitosis and partially re-replicated their DNA.

To investigate whether the EdU staining patterns we have observed indeed reflect re-replication of DNA in cells we have labeled cells with Bromodeoxyuridine (BrdU) for 18 hours that exceeds the length of a complete S-phase but is shorter than the time required for cells to undergo a complete cell cycle. We then determined the extent of BrdU incorporation into DNA using density gradients. BrdU substituted DNA is more dense (heavier) then unsubstituted DNA and the difference between unsubstituted (LL), semi-substituted (HL) and fully substituted (HH) DNA can be observed by recording the abundance of DNA in fractionated CsCl density gradients. BrdU substituted DNA was detected using anti-BrdU antibodies. As shown in Figure 6D, cells containing active Dot1L exhibited BrdU substituted DNA in fractions corresponding to HL (semi-substituted) DNA consistent with the assumption that those cells have completed a single round of replication. By contrast, cells in which Dot1L was depleted exhibited BrdU substituted DNA in both the HL and HH fractions, consistent with over-replication of a fraction of the DNA during the labeling period. Importantly, these results imply that H3K79 methylation plays a role in preventing re-replication during normal cell cycle progression.

## Discussion

The observations reported here demonstrate that H3K79Me2-containing chromatin was enriched in replication initiation events. The methylation of histone H3 on histone 79 exhibited the highest enrichment of replication initiation events observed in any single chromatin modification that was studied. It is worth noting, however, that despite the high level of enrichment in replication initiation events, not all replication initiation sites associated with H3K79 dimethylation. The association of H3K79Me2 with replication initiation sites was independent and not synergistic with other chromatin modifications. H3K79 dimethylation exhibited a wider distribution on chromatin during S-phase, but regions of chromatin that only associated with H3K79 dimethylation during S-phase were not in replication initiation events. We also observed that H3K79 dimethylation was enriched in chromatin containing a functional replicator, but was not enriched in chromatin containing a mutant replicator that could not initiate replication. Hence, H3K79 dimethylation at the human beta-globin locus replicator was associated with replicator activity. Prevention of H3K79 methylation by depletion of DOT1L did not affect the rate of DNA replication or the inter-origin distance. Importantly, however, over-replication occurred at a higher frequency following depletion of Dot1L, the sole enzyme responsible for H3K79 methylation. Together, these results demonstrate for the first time that H3K79 methylation is not required for replication initiation but rather plays a role in preventing re-replication of DNA once initiated.

Our previous studies showed that methylated CpG regions and genes undergoing moderate transcription were highly associated with replication initiation sites [3]. Other studies have also found that replication initiation sites exhibit enrichment in transcribed regions [5], G-quartets [4] and methylated CpGs [2]. Studies have also shown that methylation of H4 lysine 20 is required for genome-wide DNA replication [38], suggesting a potential mechanistic involvement of this histone modification in replication since Orc1, which exhibits an association with replication origins [39], interacts with H4K20Me2 through its BAH domain [40]. However, these studies have not identified a single histone modification that is associated with initiation of DNA replication at distinct genomic sites. Here, we have identified H3K79 methylation as a modification that is not only associated with initiation but plays a functional role in restricting replication to once per cell cycle.

Our ChIP-Seq data suggest that H3K79 dimethylation might occur in regions proximal to replication initiation sites during G1 and then expand to adjacent regions during S-phase. H3K79me2 marks again cluster with initiation sites in G2, suggesting that the S-phase specific marks, which do not associate with replication origins, might be erased and the origin-specific marks remain post-mitosis for the next cell cycle. These results imply the intriguing possibility for a mechanism to specifically and quickly remove H3K79 methylation. A precedent for an enzyme that might remove methylated histones in that way is Rph1/KDM4, which can specifically demethylate H3K36 in yeast [41]. The observed restriction of H3K79me2 containing chromatin to replication origins after S-phase might be mediated by an equivalent demethylase capable of removing methyl groups from H3K79 in non-origin regions, or by active removal of H3K79Me2 containing nucleosomes from chromatin that is not associated with replicator activity. The mechanism(s) by which potential replication origins retain H3K79 methylation whereas regions that are not associated with replication origins lose H3K79 methylation are under investigation. Regardless of the mechanism, regions of potential replication origins that can undergo initiation of DNA replication specifically retain the H3K79 methylation mark during cell division.

The most likely role played by replication origin associated DOT1L-mediated H3K79 methylation is to facilitate an interaction that marks origins that had started replication, where such a mark might prevent replication from initiating a second time. Consistent with this suggestion, H3K79Me2 associated with active but not with mutant inactive replication origins and we observed cells with late S-phase DNA content and early replication foci patterns following DOT1L depletion. In accordance, direct measurement of nucleotide incorporation also showed that Dot1 depletion resulted in partial genome over-replication, and cell cycle profiles of DOT1L depleted cells detected a larger population of cells with DNA content higher than 4N and cells with a late-S-phase DNA content. Such cells might have re-started replication of early-replicating origins without completing the replication of late-replicating origins (reflected in late S-phase DNA content and early replication patterns), or they might have completed replication of their entire genomes and skipped mitosis to re-start replication at early replication origins (reflected in DNA content greater than 4N). Taken together, over-replication of a part of the genome and early replication foci patterns in cells with late S-phase DNA content likely indicate re-replication of early replicating regions. Our observations are consistent with the hypothesis that methylation of H3K79 marks replicated regions and prevents re-initiation; when methylation is absent, cells undergo limited re-initiation of DNA replication in early replicating origins. DOT1L depleted cell populations also contain a marked fraction of cells with S-phase DNA content that are not actively replicating DNA, consistent with the suggestion that the limited re-replication we observed in the absence of H3LK79Me2 is curbed by regulatory checkpoint pathways during S-phase.

H3K79Me2 associated with an active but not with a mutant inactive replication origin, but some H3K79Me2 associated genomic regions did not exhibit strong initiation activity. These apparently disparate observations might suggest that association with H3K79Me2 plays a role in regulating replication in a subset of genomic regions, or it can reflect variations in the use of initiation sites and the fact that not all potential initiation sites start replication each cell cycle [6], [7], [14]. Currently, we have yet to identify the distinct replication initiation regions that undergo re-replication because only a fraction of cells exhibit re-replication and current methods (including NS-Seq and quantitative PCR-based measurements of nascent strand abundance) are not sufficiently sensitive to detect small alterations in the abundance of nascent strands with the precision required for drawing statistically significant conclusions. It is possible that the H3K79Me2 might mark cryptic replication initiation sites, which are capable of initiation of DNA replication but only do so under distinct conditions such as exposure to DNA damaging agents [7]. If replication does occur on such cryptic origins marked by H3K79Me2, H3K79Me2 might be available to facilitate in an interaction that will prevent re-replication from cryptic as well as constitutive origins.

Because limiting replication once per cell cycle is critical in preventing genome instability, cells employ numerous strategies to prevent re-replication [42], [43]. The mechanisms are diverse and species-specific, with components of the pre-replication licensing complex such as Cdt1 and ORC often serving as primary regulatory targets. Interestingly methylation of *Tetrahymena* histone H3K76, which is orthologous to histone H3K79 in mammals, is required for replication initiation and overexpression of the *Tetrahymena* Dot1A methylase results in over-replication [44]. Although this observation suggests that methylation of H3K76 in *Tetrahymena* achieves the opposite effect than the methylation of H3K79 in mammals, and it is yet unclear whether *Tetrahymena* H3K76 methylation associates with replication origins, both enzymes seem to be involved in regulating replication re-initiation events and thus might both be involved in processes that mark genomic regions for initiation. Another precedent for a role of chromatin modifications in regulating replication initiation is found in plants, in which histone H3 lysine 27 (H3K27) monomethyltransferases (*Arabidopsis Trithorax-Related 5* (*ATXR5*) and *ATXR6* interact with the two *Arabidopsis* proliferating cell nuclear antigen protein [45] and nuclei from *atxr5 atxr6* double mutants exhibit evidence of over-replication associated with marked decondensation of constitutive heterochromatin at chromocenters [46]. In mammals, methylation of histone H4 on lysine 20 is associated with the onset of replication licensing in G1 and the dissociation of H4K20Me1 from replication origins in S-phase is associated with prevention of re-replication [38]. Methylation of histone H4 on lysine 20 was required for initiation of DNA replication from replication origins [38], and although genome-wide analyses of replication initiation events did not exhibit a marked association with H4K20Me1 [3] it is possible that H4K20Me1 plays a role in establishing potential replication initiation patterns during the G1 phase. The temporal pattern of H3K79Me2, as reported here, is distinct from H4K20Me1. The increased frequency of apoptotic cells and cells with S-phase DNA content that do not replicate DNA suggest that the role played by H3K79Me2 in limiting re-replication is vital for cell survival and coordinated progression through the cell cycle. It is possible that the two modifications play distinct roles in regulating replication patterns and maintenance of genomic stability.

## Methods

### Cell lines and culture conditions

Human K562 cells, HCT1116 cells, U2OS cells and murine erythroleukemia cells (RL4) containing Rep-P variants were grown at 37°C in a 5% CO2 atmosphere in Dulbecco's modified Eagle medium (Invitrogen, Cat. no. 10564-011), supplemented with 10% heat-inactivated fetal calf serum. For RL4 cells containing Rep-P variants, two transgenes containing sequences from the human beta-globin Locus Control Region (HS432), the human beta-globin promoter (GloPro) driving enhanced green fluorescent protein (EGFP) and two variants of the Rep-P replicator were inserted into a single location on murine chromosome 15 in murine erythroleukemia (RL4) cells [11]. We introduced the AG1 mutation by site-directed mutagenesis. Details of the mutagenesis methods were published previously [12].

### Dot1L siRNA

HCT116 cells were transfected with 25 nM of control siRNA (Dharmacon, D-001810-10 or Dot1L siRNA (Dharmacon, L-014900-01 or Qiagen, GS84444) with DharmaFECT 2 siRNA (Dharmacon, T-2002) or RNAiMAX Transfection Reagent (Invitrogen, 13778) according to the manufacturer's protocol. Cells were transfected 2 or 3 times with a 2 to 3 days interval and harvested 72 h after the last transfection. Whole cell lysates by1XSDS loading buffer were used for western-blot with anti-Histone H3 (dimethylK79) antibody (Abcam, ab3594 or Millipore, 04-835) to verify the knockdown efficiency of dot1L siRNA.

### Nascent-strand DNA analysis

We performed nascent-strand DNA analysis as described previously [33]. DNA was extracted from asynchronous cells, denatured by boiling for 10 minutes, incubated on ice for 10 minutes, and fractionated on a neutral sucrose gradient. We collected 0.5–1 kb DNA fractions, treated them with λ exonuclease to remove non-RNA-primed genomic DNA fragments, purified them, and measured the DNA concentration using a NanoDrop 1000 (Thermo Scientific). We quantified nascent strand DNA by real-time polymerase chain reaction (PCR) in an ABI 7900 thermocycler (primers and probes used for real-time PCR are listed in Table 3). For whole genome analyses of nascent strand abundance, DNA from K562 cells was isolated using the above procedure and processed for massively parallel sequencing as previously described [3].Three independent biological replicates were sequenced to the depth of 1.4×10^8^ reads. All the data from mapping replication initiation events in K562 cells were deposited in GEO, as Series GSE28911.

### Chromatin immunoprecipitation (ChIP) analysis

We performed ChIP analyses with 1% formaldehyde-fixed K562 and RL4 cells using the Millipore ChIP assay kit (Cat. no. 17-295) following the manufacturer's protocol. Anti-H3K79Me2 antibody was from Abcam (ab3594). We analyzed ChIP samples by real-time PCR in an ABI 7900 thermocycler using primers/probes listed in Table 3.

### DNA molecular combing analysis

DNA combing analyses of replicating DNA were performed according to previously published methods [47]. Briefly, cells were pulse-labeled with 20 µM IdU (Sigma, Cat. no. I-7125) for 20 minutes and then with 50 µM CldU (MP biomedical, Cat. no. 105478) for 20 minutes before harvest. Then the cells were embedded in low-melting point agarose plugs and lysed in the plug with proteinase K lysis buffer at 50°C overnight. After agarose was digested with β-agarase (NewEngland Biolabs), DNA was combed onto silanized surfaces (Microsurfaces, Inc.) and detected with anti-IdU (BD, Cat. no. 347580), anti-CldU (Accurate Chemical, Cat. no. OBT0030), and anti-single strand DNA (Chemicon, Cat. no. MAB3034) antibodies. Images were captured with the Attovision software using the epifluorescence microscope Pathway (Becton Dickinson) and measured the signals using ImageJ (open source from National Cancer Institute, NIH) with custom-made macros [48].

### EdU staining for cell cycle analysis

Cells were cultured in 4-well chamber slides, pulse labeled with 10 µM EdU (Invitrogen) for 1 hour before harvest. EdU staining using the Click-iT EdU kit (Invitrogen) were performed according to manufacturer's protocol. Images were captured with the Attovision software using the epifluorescence microscope Pathway (Becton Dickinson) and measured with the Attovision software for DNA content by DAPI for cells with early S-phase and late-S phase EdU replication patterns.

### ChIP-Seq

Asynchronous K562 cells or fractionated G1, S and G2M K562 cells by elutriator (see [12] for elutriation details) were used for ChIP as described above. ChIP-Seq samples were sequenced using the Illumina GA II platform. The resultant sequences were aligned to the hg19 (NCBI Build 37) human reference genome using Bowtie [49]. Alignments were converted to .bam and .tdf format using SAMtools and igvtools for visualization in Broad's Integrative Genomics Viewer, http://www.broadinstitute.org/igv/ [50].

Reads per kilobase per million aligned reads (RPKM) values were calculated for each sample using 100 base genomic bins [51]. A Gaussian smoothing algorithm was applied to the bin values. To correct for sequencing biases and copy number variation, an enrichment ratio was defined as the ratio of nascent strand RPKM to control RPKM, and calculated for each 100 base bin. The H3K79Me2 and H3K4Me3 ChIP-Seq data are deposited in GEO Series GSE GSE35294 including 11 sample files from experiments in unsynchronized and cell cycle fractionated cells.

### Cesium chloride gradient for measuring BrdU density

HCT116 cells transfected with control siRNA or Dot1L siRNA were pulse-labeled with 50 µM of BrdU for 18 hours before harvest. Genomic DNA were purified and sonicated to 500–4000 bp. Genomic DNA from HCT116 cells with no BrdU incorporation and BrdU incorporation for 48 hours was used as control. 100 µg of DNA was fractionated with 6 ml CsCl (1 g/ml in TE). Samples were spun at 45000 rpm in a Ti75 rotor (Beckman) for 66 hours. Fractions of 250 µl were collected and the refractory index was measured to confirm the formation of the gradient. Same volume samples from each fraction were loaded to a positive charged nylon membrane by a Slot Blot Filtration Manifold (PR648, GE Healthcare life sciences). 0.5 ng, 5 ng, 50 ng, 500 ng and 5000 ng of total genomic DNA labeled with BrdU for 48 hours were also loaded to the membrane as standard for BrdU. BrdU were detected with anti-BrdU antibody.

## Supporting Information

Figure S1 A histogram showing the replication enrichment ratio (calculated as in Figure 1, A–E) for genomic regions as a function of their distance from the closest H3K79Me2 interaction sites. This is a box plot version of Figure 1G.
(PDF)

Figure S2 Replication initiation at JunB binding sites. A histogram showing the replication enrichment ratio for genomic regions as a function of their distance from the closest JunB interaction sites as calculated as in Figure 1G and Figure S2.
(PDF)

Figure S3 Additive effects of DNAse hypersensitivity and CpG methylation with H3K79Me2. The average replication initiation enrichment ratios of sequences exhibiting distinct chromatin features (e.g. DNAse hypersensitivity, specific histone acetylation; see Martin et al., 2011 for a list of feature tracks) were compared to the average replication initiation enrichment ratio of sequences exhibiting those features and methylation of H3K79. For each pair of chromatin features, a new double-feature track was created that contained the intersecting regions of the original features (contiguous regions were treated as one feature region). The average enrichment ratio of the double-feature track was compared to that of each of the original single-feature tracks.
(PDF)

Figure S4 A. K562 cells were fractionated by elutriation to G1, ES, MS, LS and G2M (Representative FACs profiles using propidium iodide staining before and after elutriation are shown). Cytosolic and soluble nuclear proteins were removed by hypotonic buffer with 0.5% NP40 [12]. Chromatin bound proteins were extracted by suspending the nuclei in 1× SDS-PAGE sample buffer and boiling for 5 minutes. Level of H3K79Me2 and H3K4Me3 were detected by Western blot. Ponceau S staining of Histones was used as loading controls. There are no cell cycle dependent changes for both H3K79Me2 and H3K4Me3. B. Validation of ChIP-Seq data: H3K79 dimethylation is enriched in chromatin containing a replicator. Chromatin from K562 and Jurkat cells was isolated and immunoprecipitated with an antibody directed against H3K79Me2 (Abcam, ab 3594). The abundance of sequences from the human beta-globin locus was analyzed in DNA isolated from immunoprecipitated chromatin by real-time PCR as described [12] using primers/probe combinations listed in Table 3. The locations of primers/probes in the beta-globin locus are illustrated under the histogram and the boundaries of the two replicators (Rep-P and Rep-I) within the initiation region (IR) are shown [33], [34]. H3K79Me2 containing chromatin were markedly enriched in sequences amplified by primers designated bG59.9 and bG61.3, located within the Rep-P replicator, and the enrichment was very high in sequences amplified by the primer pair AG, located at an asymmetric region essential for replicator activity.
(PDF)

Figure S5 Example images of fiber analyses of DNA replication. Raw images were shown for DNA combing analyses in Figure 5 and Figure S2. Cells were labeled sequentially with ldU and CIdU, then the DNA was stretched on a silanized microscope coverslip, and visualized with antibodies against DNA containing ldU (green replication tracks) and CldU (red replication tracks)(top images in both A and B). DNA fibers were detected by anti-single strand antibody (bottom image in both A and B).The white vertical lines are examples how replication signals are defined and the distance between them are measured by Image J with a custom-made macro.
(PDF)

Figure S6 Depletion of H3K79 methytansferase *DOT1*L does not change replication initiation rates. HCT116 cell treatments and DNA combing analyses were described in Figure 4. A. A histogram of the distribution of inter-origin distance measured in DNA fibers from cells transfected with scrambled siRNA. B. A histogram of the distribution of inter-origin distance measured in DNA fibers from cells transfected with siRNA targeting *DOT1*L.
(PDF)

Figure S7 Effects of Dot1L depletion on cell cycle progression inU2OS cells. U2OS cells were transfected with control or *DOT1L* siRNA once or twice with a 3 day interval and collected for FACs 3 days after the last transfection. EdU were added to cells for 45 minutes before harvesting cells. Click-iT EdU kit from Invitrogen was used to detect replicating cells and DAPI was used to determine DNA content. A. Representative cell cycle profile for cells transfected with control siRNA or Dot1L siRNA 3 days after the first transfection (top panel) and 3 days after the second transfection (lower panel). B. A summary histogram of the cell cycle distribution of U2OS cells 3 days after the second transfection with control siRNA or Dot1L siRNA.
(PDF)

Table S1 Cell lines used in this work. A list of cell lines and their backgrounds, as well as reasons being chosen.
(PDF)

Table S2 Fraction of cells at various stages of the cell cycle following depletion of Dot1L from HCT116 and U2OS cells.
(PDF)
