# Correction: Methylation of Histone H3 on Lysine 79 Associates with a Group of Replication Origins and Helps Limit DNA Replication Once per Cell Cycle

**DOI:** 10.1371/annotation/2b2d77ee-6d39-4685-b2e5-ebba0b793902

**Published:** 2013-07-30

**Authors:** Haiqing Fu, Alika K. Maunakea, Melvenia M. Martin, Liang Huang, Ya Zhang, Michael Ryan, RyangGuk Kim, Chii Meil Lin, Keji Zhao, Mirit I. Aladjem

There was an error in the last paragraph of the Discussion section. The third and fourth sentences should read:

"Interestingly methylation of Trypanosoma brucei H3K76, which is orthologous to histone H3K79 in mammals, is required for replication initiation and overexpression of the Trypanosoma Dot1A methylase results in over-replication[44]. Although this observation suggests that methylation of H3K76 in Trypanosoma achieves the opposite effect than the methylation of H3K79 in mammals, and it is yet unclear whether Trypanosoma H3K76 methylation associates with replication origins, both enzymes seem to be involved in regulating replication re-initiation events and thus might both be involved in processes that mark genomic regions for initiation. "

