# Supplementary figures and images for: Methylation of Histone H3 on Lysine 79 Associates with a Group of Replication Origins and Helps Limit DNA Replication Once per Cell Cycle
Source: PLoS Genet. 2013 Jun 6;9(6):e1003542. doi: 10.1371/journal.pgen.1003542 (PMC3674996; doi:10.1371/journal.pgen.1003542)

Figure S1

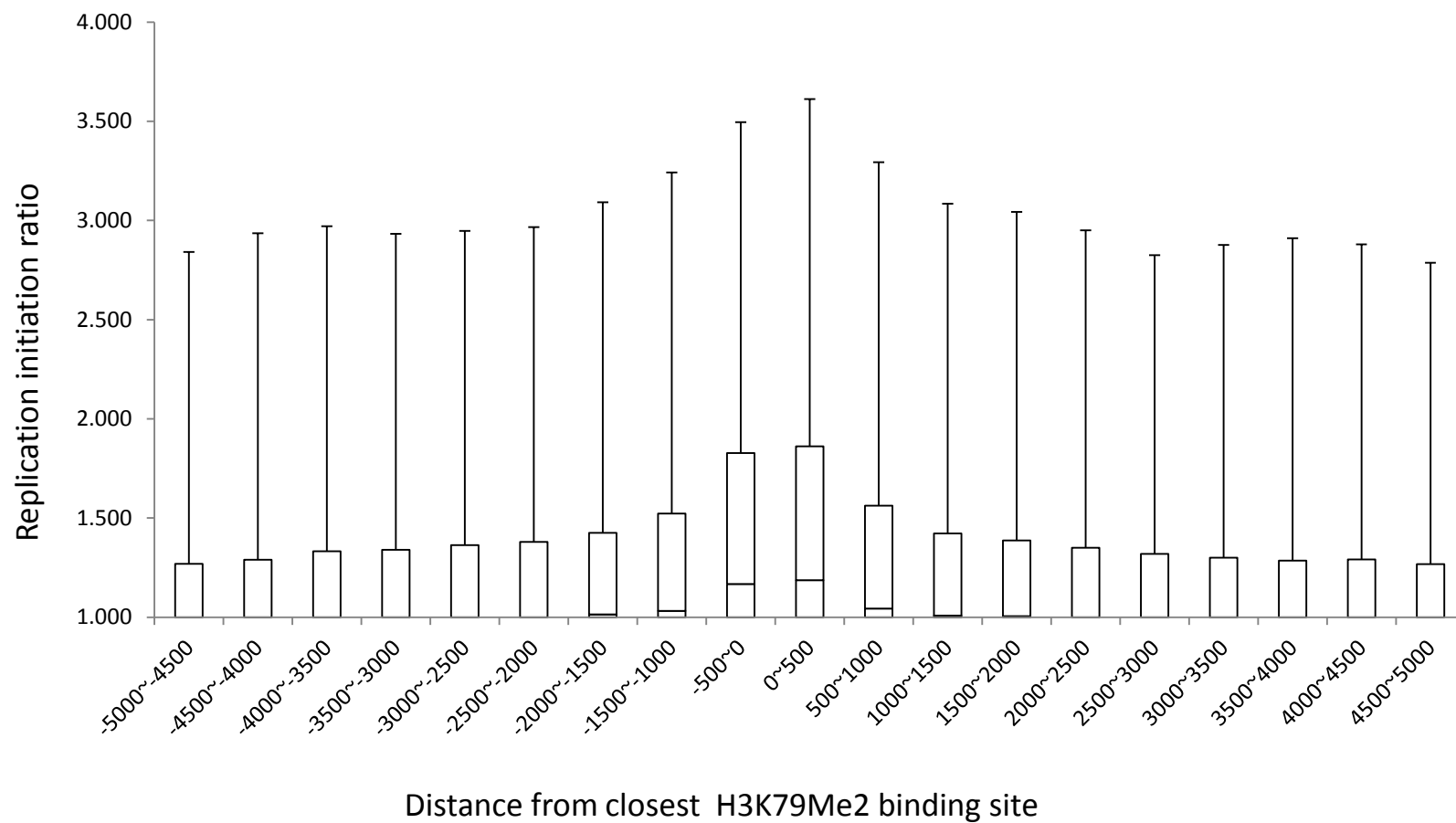

Supplement: Figure S1 — A histogram showing the replication enrichment ratio (calculated as in Figure 1, A–E) for genomic regions as a function of their distance from the closest H3K79Me2 interaction sites. This is a box plot version of Figure 1G. (PDF) [file pgen.1003542.s001.pdf]

Figure S2

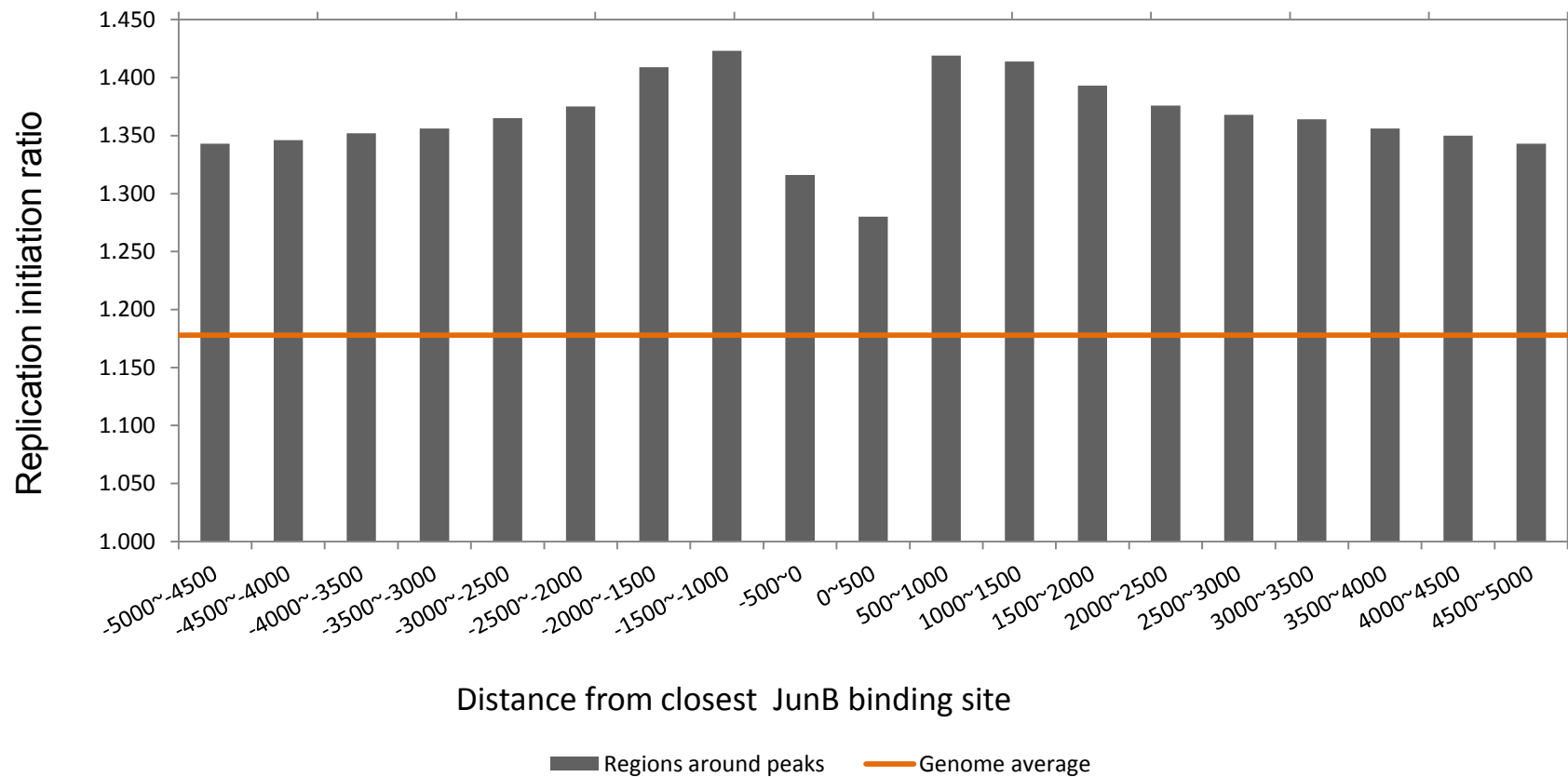

Supplement: Figure S2 — Replication initiation at JunB binding sites. A histogram showing the replication enrichment ratio for genomic regions as a function of their distance from the closest JunB interaction sites as calculated as in Figure 1G and Figure S2. (PDF) [file pgen.1003542.s002.pdf]

Figure S3

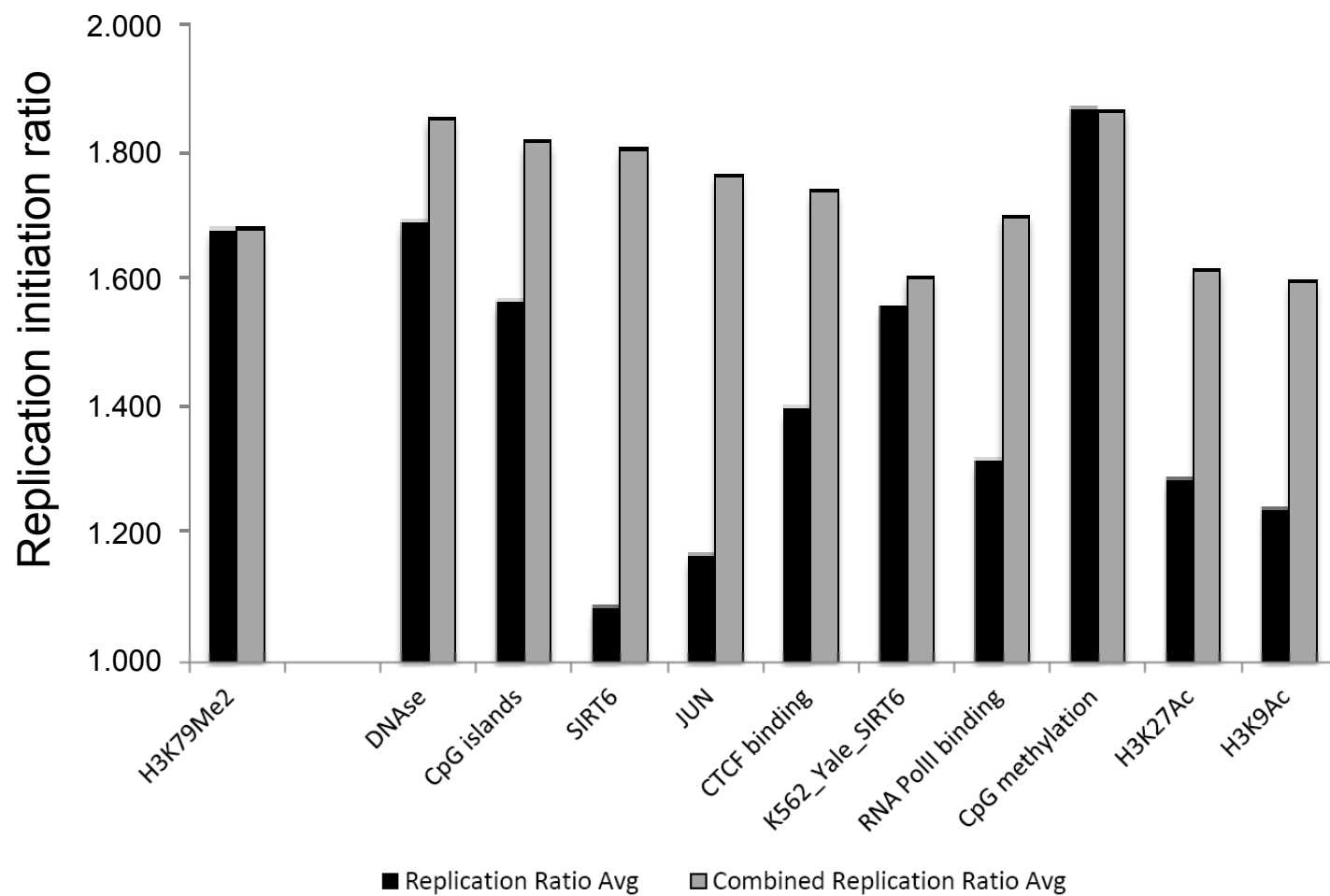

Supplement: Figure S3 — Additive effects of DNAse hypersensitivity and CpG methylation with H3K79Me2. The average replication initiation enrichment ratios of sequences exhibiting distinct chromatin features (e.g. DNAse hypersensitivity, specific histone acetylation; see Martin et al., 2011 for a list of feature tracks) were compared to the average replication initiation enrichment ratio of sequences exhibiting those features and methylation of H3K79. For each pair of chromatin features, a new double-feature track was created that contained the intersecting regions of the original features (contiguous regions were treated as one feature region). The average enrichment ratio of the double-feature track was compared to that of each of the original single-feature tracks. (PDF) [file pgen.1003542.s003.pdf]

A

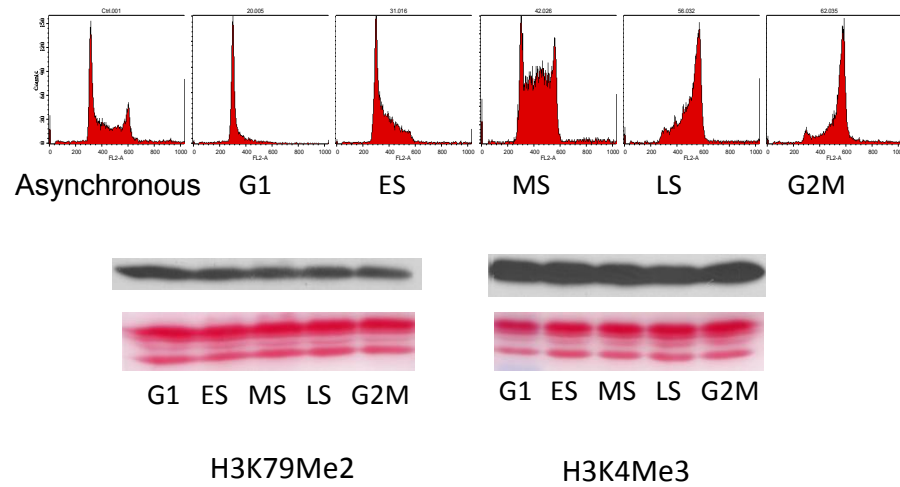

Figure S4

B

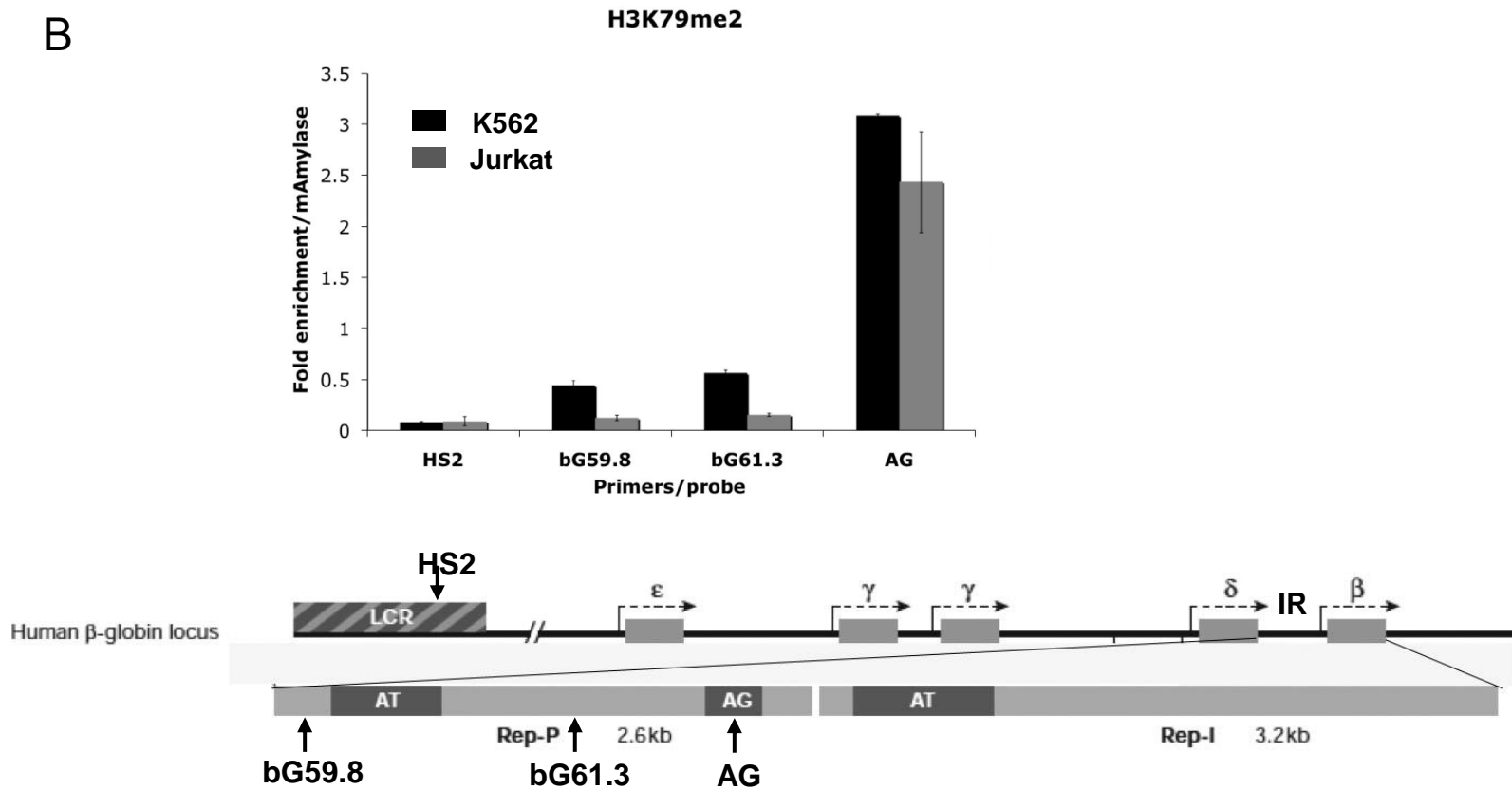

Supplement: Figure S4 — A. K562 cells were fractionated by elutriation to G1, ES, MS, LS and G2M (Representative FACs profiles using propidium iodide staining before and after elutriation are shown). Cytosolic and soluble nuclear proteins were removed by hypotonic buffer with 0.5% NP40 [12]. Chromatin bound proteins were extracted by suspending the nuclei in 1× SDS-PAGE sample buffer and boiling for 5 minutes. Level of H3K79Me2 and H3K4Me3 were detected by Western blot. Ponceau S staining of Histones was used as loading controls. There are no cell cycle dependent changes for both H3K79Me2 and H3K4Me3. B. Validation of ChIP-Seq data: H3K79 dimethylation is enriched in chromatin containing a replicator. Chromatin from K562 and Jurkat cells was isolated and immunoprecipitated with an antibody directed against H3K79Me2 (Abcam, ab 3594). The abundance of sequences from the human beta-globin locus was analyzed in DNA isolated from immunoprecipitated chromatin by real-time PCR as described [12] using primers/probe combinations listed in Table 3. The locations of primers/probes in the beta-globin locus are illustrated under the histogram and the boundaries of the two replicators (Rep-P and Rep-I) within the initiation region (IR) are shown [33], [34]. H3K79Me2 containing chromatin were markedly enriched in sequences amplified by primers designated bG59.9 and bG61.3, located within the Rep-P replicator, and the enrichment was very high in sequences amplified by the primer pair AG, located at an asymmetric region essential for replicator activity. (PDF) [file pgen.1003542.s004.pdf]

Figure S5

A

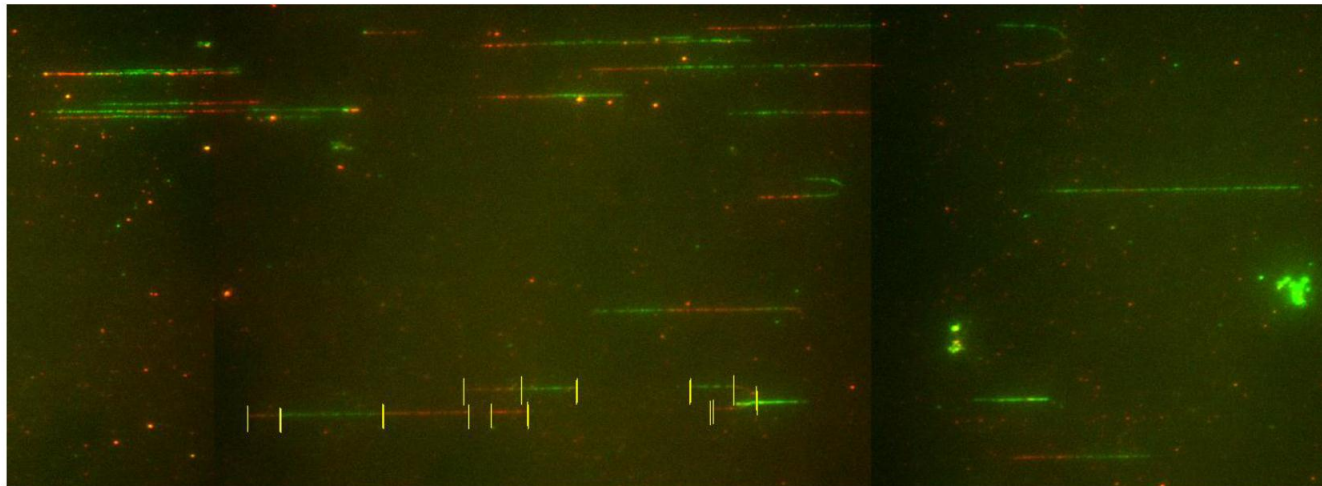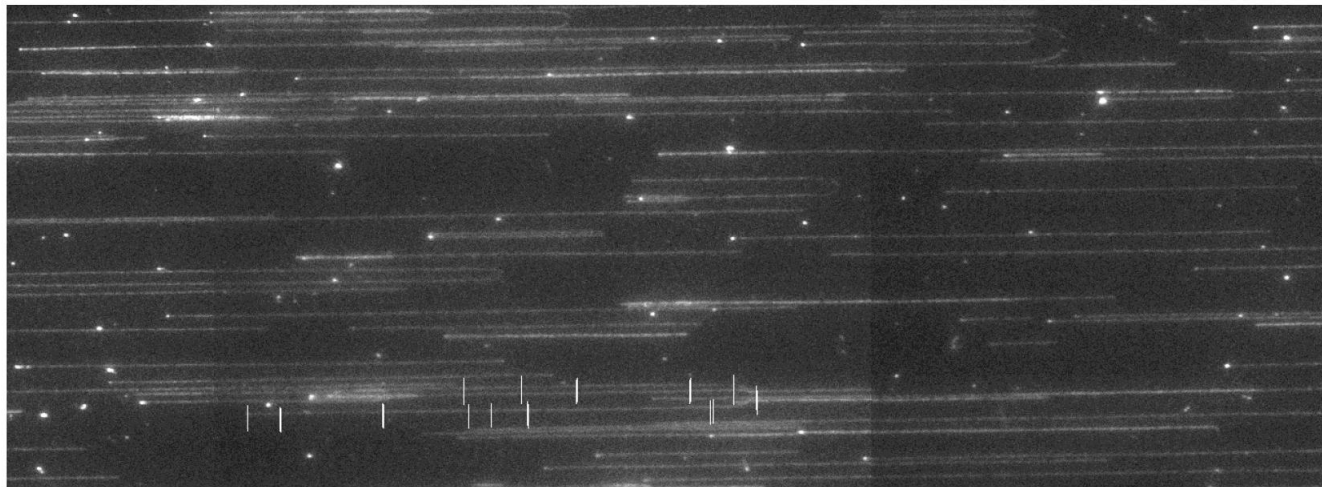

100kb

## Supplemental Figure 5 (Cont)

B

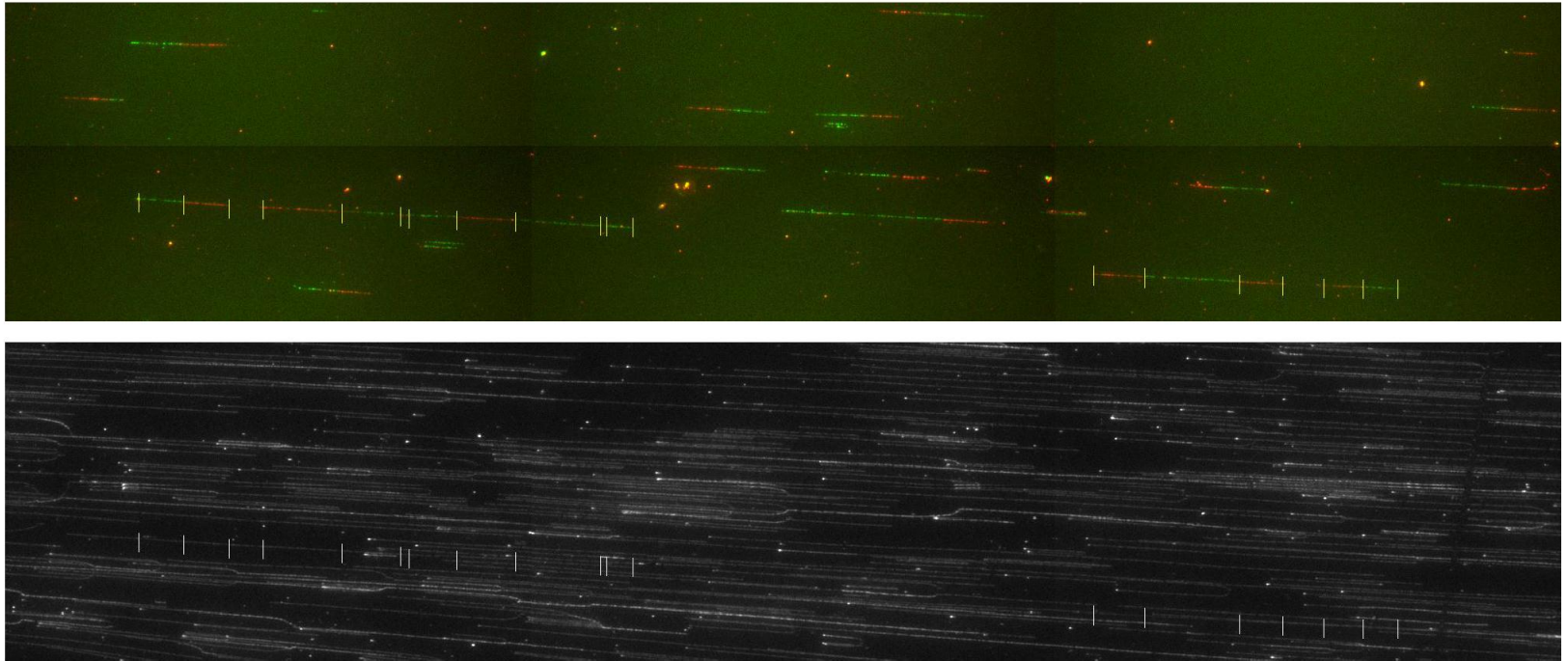

100kb

Supplement: Figure S5 — Example images of fiber analyses of DNA replication. Raw images were shown for DNA combing analyses in Figure 5 and Figure S2. Cells were labeled sequentially with ldU and CIdU, then the DNA was stretched on a silanized microscope coverslip, and visualized with antibodies against DNA containing ldU (green replication tracks) and CldU (red replication tracks)(top images in both A and B). DNA fibers were detected by anti-single strand antibody (bottom image in both A and B).The white vertical lines are examples how replication signals are defined and the distance between them are measured by Image J with a custom-made macro. (PDF) [file pgen.1003542.s005.pdf]

Figure S6

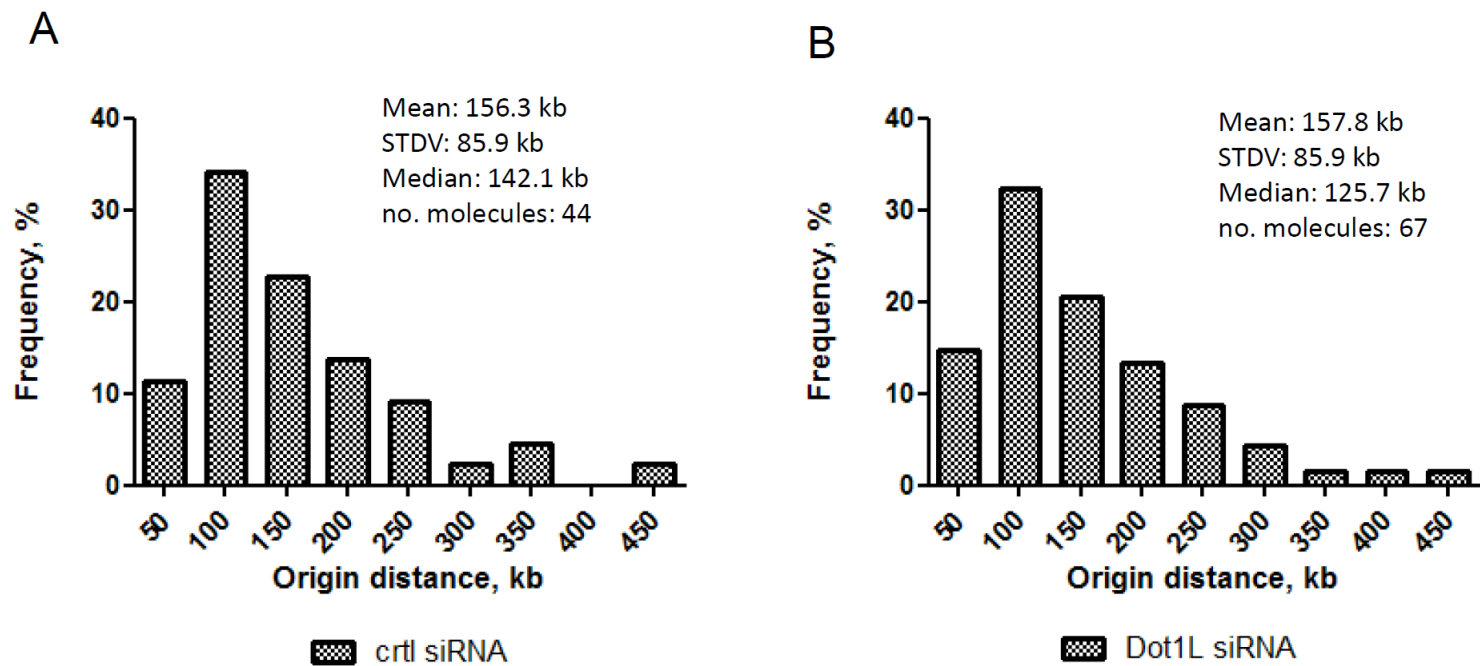

Supplement: Figure S6 — Depletion of H3K79 methytansferase DOT1L does not change replication initiation rates. HCT116 cell treatments and DNA combing analyses were described in Figure 4. A. A histogram of the distribution of inter-origin distance measured in DNA fibers from cells transfected with scrambled siRNA. B. A histogram of the distribution of inter-origin distance measured in DNA fibers from cells transfected with siRNA targeting DOT1L. (PDF) [file pgen.1003542.s006.pdf]

Figure S7

A

3 days after 1st  
transfection

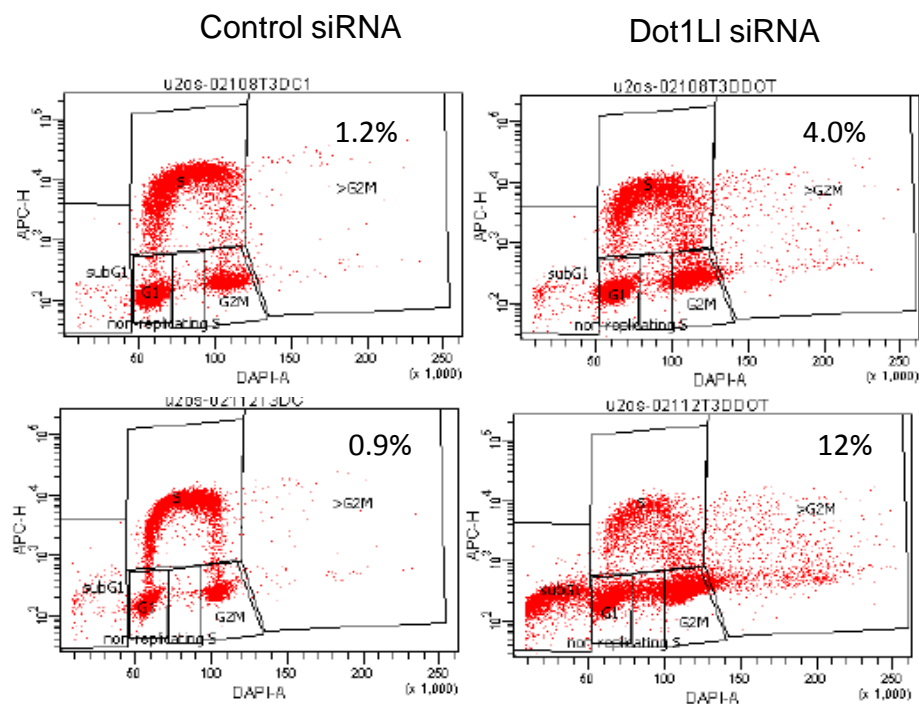

B

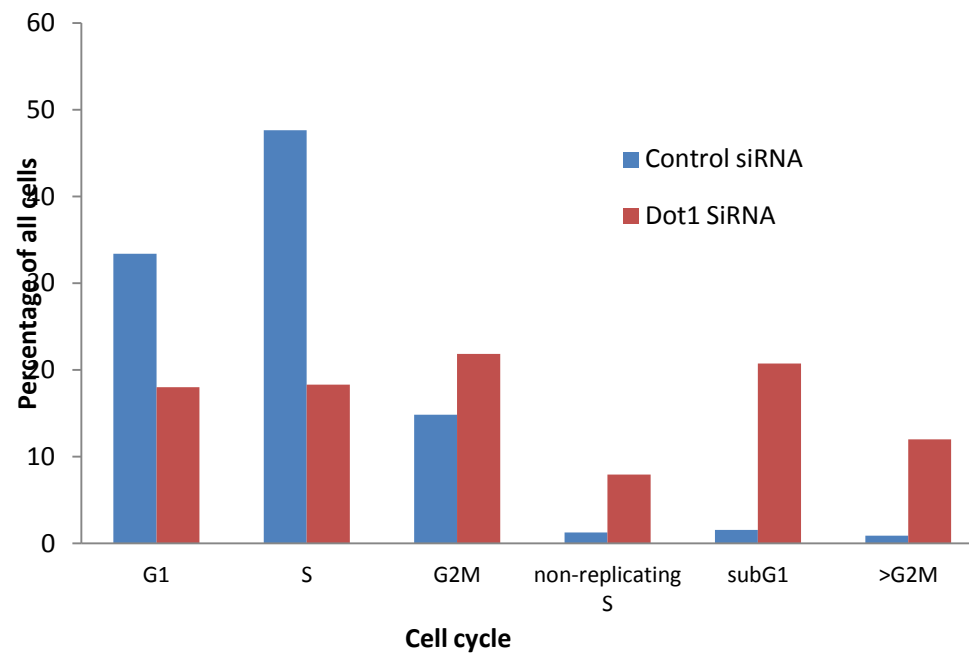

Supplement: Figure S7 — Effects of Dot1L depletion on cell cycle progression inU2OS cells. U2OS cells were transfected with control or DOT1L siRNA once or twice with a 3 day interval and collected for FACs 3 days after the last transfection. EdU were added to cells for 45 minutes before harvesting cells. Click-iT EdU kit from Invitrogen was used to detect replicating cells and DAPI was used to determine DNA content. A. Representative cell cycle profile for cells transfected with control siRNA or Dot1L siRNA 3 days after the first transfection (top panel) and 3 days after the second transfection (lower panel). B. A summary histogram of the cell cycle distribution of U2OS cells 3 days after the second transfection with control siRNA or Dot1L siRNA. (PDF) [file pgen.1003542.s007.pdf]
