# Supplementary material for: Methylation of Histone H3 on Lysine 79 Associates with a Group of Replication Origins and Helps Limit DNA Replication Once per Cell Cycle
Source: PLoS Genet. 2013 Jun 6;9(6):e1003542. doi: 10.1371/journal.pgen.1003542 (PMC3674996; doi:10.1371/journal.pgen.1003542)
Supplement: Table S1 — Cell lines used in this work. A list of cell lines and their backgrounds, as well as reasons being chosen. (PDF) [file pgen.1003542.s008.pdf]

Table S1. Cell lines used in this work.

Name of cell line	Backgrounds and/or reasons being used
**K562**	Human erythroleukemia cells that express the gamma globin in which the beta-globin locus replicates early during S phase. This cell line was used to map the association between H3K79Me2 and replication initiation because /1/it was used for numerous replication-related studies including whole genome origin mapping data; /2/ChIP-Seq data delineating biding sites of many histone modifications are available for K562 cells; /3/ the cells are amenable to fractionation according to cell cycle stages using centrifugal elutriation.
**Jurkat**	Human T-cell leukemia cells that do not express any gene within the beta-globin locus and although they start replication from the same region within beta globin locus as k562 cells, they replicate the locus late during S-phase. This cell line was used to test the association between H3K79Me2 and late-replicating replication initiation site.
**HCT116**	Human colon cancer cells that replicate the beta globin locus late during the S-phase. This cell line was used in studies requiring siRNA mediated depletion of dot1L due to the low efficiency in K562 cells.
**U2OS**	Human osteosarcoma cells used for test the phenotype observed following Dot1L depletion in U2OS cells.
**RL4 MEL**	Murine erythroleukemia cells were used in studies that required insertion of replicator variants (functional and mutants) into ectopic chromatin. An insertion site in RL4 MEL facilitate the insertion of transgene cassettes in a consistent location. Replication initiation from human sequences inserted into single copy transgenes can be measured with minimal background using sequences from the murine beta globin locus as controls.
