# Supplementary material for: Methylation of Histone H3 on Lysine 79 Associates with a Group of Replication Origins and Helps Limit DNA Replication Once per Cell Cycle
Source: PLoS Genet. 2013 Jun 6;9(6):e1003542. doi: 10.1371/journal.pgen.1003542 (PMC3674996; doi:10.1371/journal.pgen.1003542)
Supplement: Table S2 — Fraction of cells at various stages of the cell cycle following depletion of Dot1L from HCT116 and U2OS cells. (PDF) [file pgen.1003542.s009.pdf]

Table S2. Effects of Dot1L depletion on cell cycle progression in HCT116 cells (data corresponding to Figure 5 and Figure S7)

	HCT 116 cells		U2OS cells	
	Control siRNA	Dot1L siRNA	Control siRNA	Dot1 SiRNA
G1	29.15	30.1	33.4	18
S	52	40.45	47.65	18.3
G2M	16.75	18.25	14.85	21.85
non-replicating S	0.6	3	1.25	7.95
subG1	0.9	8.7	1.55	20.75
>G2M	1.1	2.55	0.9	12
